# Is Pelvic Floor Muscle Resting Activity Associated with Pelvic and Genital Pain, Dyspareunia, and Pelvic Floor Muscle Contraction? A Cross-Sectional Study of Women with Endometriosis

**DOI:** 10.1007/s00192-025-06190-2

**Published:** 2025-07-02

**Authors:** Rakel Gabrielsen, Kari Bø, Marie Ellström Engh, Merete Kolberg Tennfjord

**Affiliations:** 1 Department of Obstetrics and Gynecology Nordbyhagen, Akershus University Hospital, Nordbyhagen, Norway; 2 Faculty of Medicine, University of Oslo, Oslo, Norway; 3 Department of Sports Medicine, Norwegian School of Sport Sciences, Oslo, Norway; 4 Department of Health and Exercise, Kristiania University College, Oslo, Norway

**Keywords:** Dyspareunia, Endometriosis, Pelvic and genital pain, Pelvic floor muscle activity, Pelvic floor muscle resting activity

## Abstract

**Introduction and Hypothesis:**

A link between pelvic and genital pain, dyspareunia, and increased pelvic floor muscle (PFM) tone is an area of controversy. Additionally, it has been postulated that increased PFM tone can limit the ability to further activate the PFM. We aimed to investigate the association between PFM resting activity and pelvic and genital pain and dyspareunia, and whether there is an association between PFM resting activity and activation during attempts at PFM maximal voluntary contractions (MVCs) in women with endometriosis.

**Methods:**

This cross-sectional study included 80 women with endometriosis and pelvic and genital pain. An electronic questionnaire included background information, pelvic and genital pain (numeric rating scale 0–10) and questions about location and concerns of dyspareunia. Associations between variables were analyzed using multiple linear regression. PFM resting activity was registered as the mean microvolt (μV) during rest before and between five voluntary MVCs of the PFM.

**Results:**

Mean age was 29 years (SD 6.2), and 9 (11%) were parous. No significant association between resting activity, pelvic and genital pain or location and concerns of dyspareunia was found. A significant positive association between PFM resting activity and activation during attempts at MVCs of the PFM (β = 0.130, *p* = 0.009, 95% CI = 0.034–0.229) was found.

**Conclusion:**

No association was found between PFM resting activity and pelvic and genital pain or location and concerns of dyspareunia. Contrary to the hypothesis, higher PFM resting activity resulted in more activation of the PFM during attempts at MVCs.

**Supplementary Information:**

The online version contains supplementary material available at 10.1007/s00192-025-06190-2.

## Introduction

Persistent pelvic and genital pain and dyspareunia are common complaints among women with endometriosis [1]. A connection between pelvic and genital pain, dyspareunia and increased pelvic floor muscle (PFM) tone has been suggested [2]. However, it remains unclear whether PFM tone plays a role in the development of these pain conditions or whether the conditions lead to increased tone. PFM tone is defined as the state of the muscle, usually defined by its resting tension, and is clinically determined by resistance to passive movement [3]. PFM tone can be classified as normal, decreased, or increased [4]. A comparison with established reference values is required to confirm that PFM tone is increased beyond “normal”; however, such reference values are not currently available [5]. Further, muscle tone comprises active components (muscle activity), and passive components (viscoelastic tissue properties) [3, 6]. These terms are often used interchangeably and can create confusion in clinical practice and research, as they refer to distinct aspects of muscle tone. Surface electromyography (sEMG) is commonly used among physical therapists to assess the myoelectrical activity of the PFM at rest with either a probe with a sensor or with external electrodes, which reflects the active components of resting tone, referred to as resting activity [7]. Among women with endometriosis, increased PFM muscle tone has been found compared with those without endometriosis [8]. However, in a study by Dos Bispo et al. in 2016, one physiotherapist assessed the passive components of tone through vaginal palpation, and therefore, the responsiveness, reliability, and validity of these findings can be questioned [9]. Although the same tester performed all examinations from the latter study, and digital palpation has shown good intraobserver reliability [10], it has low interrater reliability/test–retest reliability, making it less suitable for research purposes [11]. Additionally, we lack a reliable and validated palpation scale for PFM stiffness [9].

A recent meta-analysis found no convincing evidence for increased PFM tone and pelvic pain when using reliable and valid measurement devices [12]. Similarly, a systematic review highlighted that research on PFM tone and pain had been constrained by inconsistencies in terminology, study design, and measurement method [5].

Some authors have proposed that manual treatment of the PFM (such as massage) is the most effective approach for addressing increased PFM pain. They claim that pelvic floor muscle training (PFMT) may be contraindicated for certain individuals [13]. It has also been suggested that increased tone might impair the ability to perform an MVC and “down training” of the PFM to restore a normal tone of the PFM. To the best of our knowledge, no studies have investigated whether there is a link between PFM muscle resting activity and the ability to further increase PFM activity.

The aims of this study were two-fold:

1. To investigate the association between PFM resting activity, pelvic and genital pain and location, and concerns related to dyspareunia in women with endometriosis.
2. To investigate whether there is an association between PFM resting activity and activation of the PFM during attempts at MVC.

We hypothesized that increased resting activity might be associated with more pelvic and genital pain and dyspareunia in women with endometriosis and that increased PFM resting activity might be associated with less activation during MVC.

## Materials and Methods

### Study Design and Setting

This cross-sectional study uses baseline data from 80 women diagnosed with endometriosis participating in an assessor-blinded, two-arm, parallel-group randomized trial conducted between January 2022 and January 2023.

### Participants

Inclusion criteria were women aged 18–45 with endometriosis confirmed by laparoscopy and presenting with pelvic and genital pain ≥ 4/10 at its worst measured on a numeric rating scale (NRS) [14]; ability to understand and speak Norwegian language. Exclusion criteria were severe pathology, including psychiatric disorders that required admission to the hospital; personality disorders diagnosed by specialists; pregnancy or childbirth in the last 12 months or breastfeeding; and intra-abdominal or vaginal surgery. Women who had received any type of PFM injections, hormonal therapy, or PFM treatment from a physical therapist within the past 4 to 6 months were also excluded. None of the participants had prior acquaintance with the tester.

Women were recruited from the Departments of Obstetrics and Gynecology at two public university hospitals and by advertising on social media and through the Norwegian endometriosis association during 2021–2023. Upon contact, they received written information about the project, and if they were eligible for the project, a phone call was made, including information about the PFM assessments. They also received an e-mail with a weblink including an electronic questionnaire with demographics in addition to questions on self-reported pelvic and genital pain and a questionnaire about location and concerns related to dyspareunia. All women gave their informed consent to participation. The study followed the STrengthening the Reporting of OBservational studies in Epidemiology guidelines for cross-sectional studies [15] (Supplementary Fig. 1).

### Data Collection

Pelvic and genital pain were measured and registered on a numeric rating scale (NRS) where 0 indicated no pain and 10 indicated the worst imaginable pain over the course of 1 month. This scale has shown excellent test–retest reliability in other patient populations of inflammatory conditions [16], it is easy to use, sensitive to change, and able to monitor pain and treatment effectiveness [14]. To visualize the pelvic and genital area, a pain body map on which participants marked the locations of their pain was included.

With a lack of standardized outcome measures, location and concerns related to dyspareunia were evaluated using a questionnaire developed at a treatment center for advanced endometriosis Sahlgrenska University Hospital, Gothenburg, Sweden (Table 1), the questionnaire included questions about location (deep or superficial) and four questions about concerns related to dyspareunia (Table 1). The symptom scores were dichotomized into never/rarely/sometimes or often/always. The questionnaire has no total score; each question was analyzed individually. The questionnaire has not yet been validated.

**Table 1:** Questions about location and concerns related to dyspareunia

How often in the past 4 weeks	Not applicable	Never	Rarely	Sometimes	Often	Always
1. Have you felt deep pain during or after sexual intercourse?						
2. Have you felt superficial pain during or after sexual intercourse?						
3. Have you been concerned about having sexual intercourse because of pain?						
4. Have you avoided sexual intercourse because of the pain?						
5. Have you felt guilty about not having intercourse?						
6. Have you been frustrated that you could not enjoy sexual intercourse?						

The PFM assessments were conducted by a specialist woman’s health physical therapist with over 10 years of experience. The physical therapist was blinded regarding the background data collected through the electronic questionnaires prior to the PFM assessment.

#### Ability to Contract the Pelvic Floor Muscle

Visual observation and vaginal palpation were used to assess the ability to contract and relax the PFM after contraction, as recommended [17, 18]. The participants were instructed to breathe in and out while relaxing their PFM, avoiding any voluntary PFM activity during the resting period. A correct PFM contraction was defined as an inward movement of the perineum and a squeeze around the pelvic openings [19]. The PFM assessment was conducted with the participants lying supine on a bench, with hips and knees in flexion and feet flat on the bench. Participants were asked to empty their bladder before the examination, following clinical guidelines for PFM assessments [4].

#### Pelvic Floor Muscle Activity

**Fig. 1:**
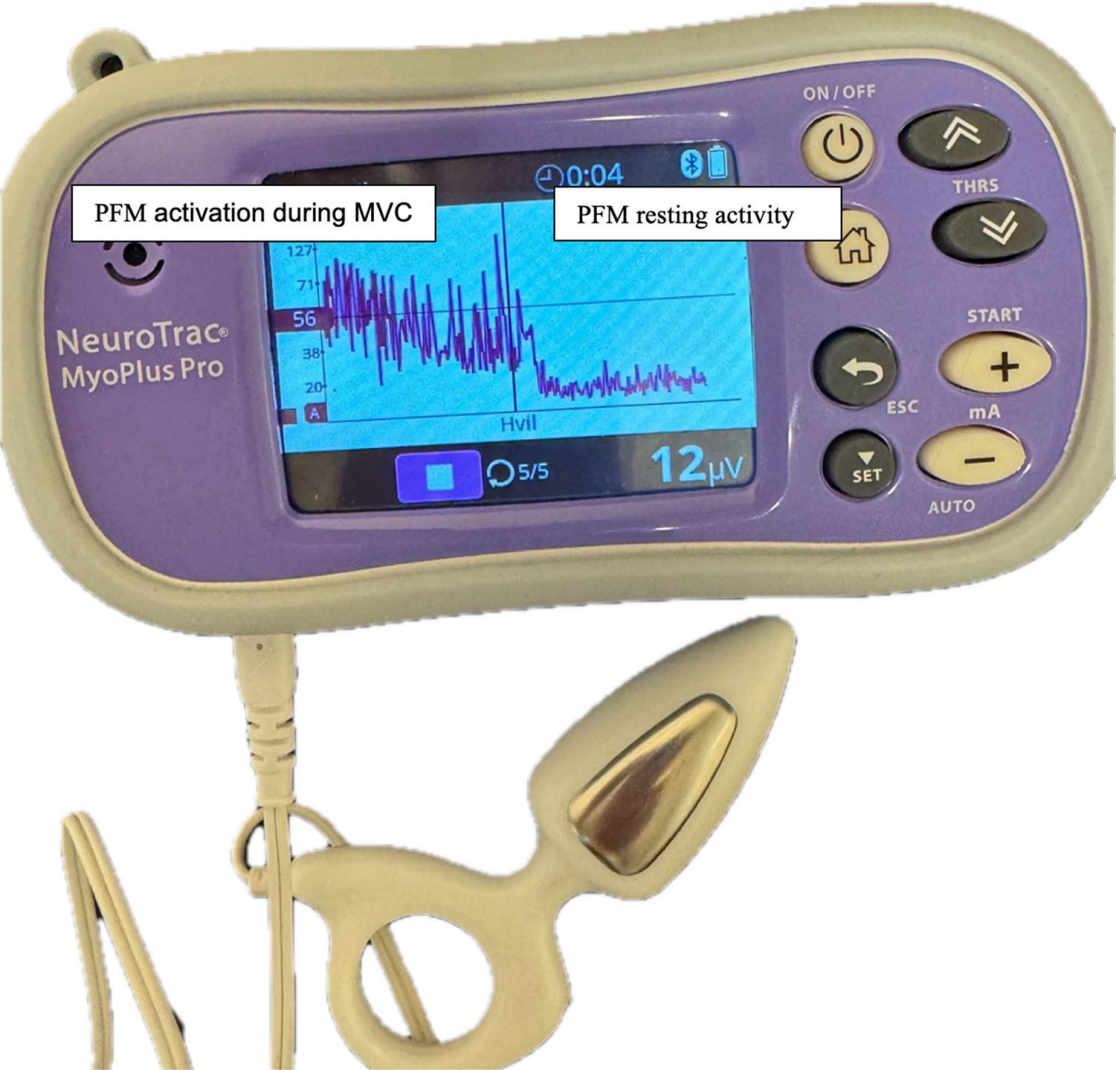
Surface electromyography, NeuroTrac MyoPlus Pro Quintet, Bergen, Norway with an Anuform probe and example of curves of pelvic floor muscle (PFM) resting activity and activation during maximal voluntary contraction (mean of five contractions)

The same physical therapist performed the PFM activity measurements. PFM resting activity and activation during attempts at MVCs of the PFM were assessed by intravaginal sEMG registered in microvolts (μV, NeuroTrac MyoPlus) Pro Quintet, Bergen, Norway was used to measure PFM activity (Fig. 1). An anal probe (Anuform, Quintet, Bergen, Norway) with a transverse diameter of 25 mm and two stainless steel lateral electrodes (35 × 15 mm) was used for assessment owing to pain experienced with the periform probe. The probe was inserted vaginally with the electrodes placed in the 3 and 9 o’clock positions and the ring in a vertical position. The sEMG reference lead wire with an adhesive electrode was placed near the wrist on the right hand to improve the sEMG signal quality [20]. PFM resting activity was registered in a supine position with the instruction to relax the PFM and calculated by averaging the microvolt (μV) readings during the rest periods before and between PFM contractions. The calculation of PFM resting activity excluded the first second of each measurement to eliminate the initial spikes from the first contraction attempt and the early instability during relaxation (NeuroTrac® MyoPlus PRO Operation Manual). Activation during attempts at MVC was reported as the mean of 5 PFM contractions (μV).

### Statistical Analyses

Data were analyzed using SPSS 29 (IBM SPSS Statistics for Windows, Version 29.0, IBM Corp). Outliers and extreme values were identified and removed based on thresholds observed in histograms and box plots. Specifically, extreme values that exceeded 1.5 times the interquartile range (IQR) above the upper quartile or below the lower quartile were excluded from the analysis. Background variables are reported as mean and standard deviation (SD) or numbers with percentages. Multicollinearity between “deep pain during sexual intercourse” and “superficial pain during sexual intercourse” (correlation 0.542, *p* = 0.001) was found. Similarly, for the questions “had a bad conscience for not wanting to have intercourse” and “avoided intercourse because of pain” (correlation 0.447, *p* = 0.001). Based on clinical reasoning, superficial pain during sexual intercourse was included (termed “superficial dyspareunia”) and “avoided intercourse because of pain” was included in the analysis in addition to “concerns about having sexual intercourse” and “been frustrated that you cannot enjoy intercourse.”

A multiple regression analysis was used to investigate the association between resting activity as the dependent variable and the independent variables pelvic and genital pain, and location and concerns related to dyspareunia, controlling for age and parity in the analysis. To investigate the association between PFM resting activity and activation during attempts at PFM MVC, a multiple linear regression analysis was conducted with PFM resting activity as the dependent variable and mean activation during five attempts at MVC as the independent variable. Age and parity were considered potential confounding variables and included in the analysis to ensure that these variables did not confound the association between PFM resting activity and the mean MVC.

The regression coefficient (B) and 95% confidence interval (CI) were used to describe the strength and precision of the above associations. A significance level of *p* < 0.05 was considered significant.

## Results

### Participant Characteristics

Out of 168 women assessed for eligibility, 80 were included in the study. The reasons for nonparticipation were not meeting the inclusion criteria (*n* = 72), not being interested in participating (*n* = 5), and other unknown personal reasons (*n* = 10). In addition, one was excluded owing to extreme values that exceeded 1.5 times the IQR. Table 2 shows the background data of the women included.

**Table 2:** Baseline characteristics of the women

Variables	Total sample (*N* = 80)
Age, years, mean (SD)	29 (6.2)
Body mass index, kg/m^2^, mean (SD)	25.2 (5)
Civil status, *n* (%)	
Married/cohabiting	44 (53)
Single	38 (46)
Parous (yes), *n* (%)	9 (11)
Ethnicity, *n* (%)	
European	76 (94)
African	1 (1)
Asian	2 (2)
Don’t know	2 (2)
Education, *n* (%)	
> 4 years/higher education	30 (40)
Bachelor	16 (22)
High school	22 (30)
Primary school/other	5 (7)
Employment, *n* (%)	
Paid work (full-time/part-time)	63 (76)
Sick leave	15 (18)
Disability leave	2 (3)
Physically strenuous work (yes)	46 (57)
Alcohol consumed, *n* (%)	
< 1 unit per/week	37 (46)
> 1–4 units per week	33 (40)
5–10 units per week	11(2)
Snuffing (yes), *n* (%)	23 (23)
Smoking (yes), *n* (%)	0
Time since diagnoses, mean (SD)	5 (3.4)
Duration of abdominal/menstrual pain, *n* (%)	
0–4 years	1 (1)
5–10 years	24 (30)
11–15 years	26 (32)
16–20 years	16 (20)
> 21 years	14 (17)
Dyspareunia, *n* (%)	
Deep pain	13 (16)
Superficial pain	42 (52)
Pelvic and genital pain, numeric rating scale, mean (SD)	7.5 (2.05)

*SD* standard deviation

All the women were able to contract their PFM. The mean resting activity measured with sEMG was 17.9 (SD 16) μV, and the mean activity during MVC was 46.9 (SD 28) μV.

Data from 75 of the women were analyzed for the PFM measurements. Five of the women could not insert the probe owing to pain and were therefore excluded from the sEMG measurements. The mean NRS for pelvic and genital pain among these five women was 7.17 (SD 3), indicating severe pain.

No association was found among these variables when assessing the relationship between sEMG resting activity, pelvic and genital location, and concerns related to dyspareunia (Table 3).

**Table 3:** Multiple linear regression analysis showing the association between pelvic floor muscle (PFM) resting activity measured with surface electromyography (sEMG), pelvic and genital pain, location, and concerns related to dyspareunia, *n* = 75^a^

Reported symptoms	Unadjusted estimates		Adjusted estimates	
	B (95% CI)	*p* value	B (95% CI)	*p* value
Pelvic and genital pain NRS 0–10	−0.020 (−1.498 to 1.449)	0.979	−0.117 (−2.217 to 0.775)	0.339
Superficial dyspareunia^b^	2.083(−3.759 to 7.925)	0.479	6.221 (−3.414 to 15.013)	0.199
Concerns about having sexual intercourse	1.049 (−6.468 to 8.566)	0.782	4.697 (−5.831 to 15.225)	0.373
Avoided intercourse because of the pain	5.335 (−0.981 to 11.652)	0.097	3.370 (−5.263 to 12.002)	0.222
Been frustrated not being able to enjoy intercourse	−2.567 (−7.505 to 2.370)	0.303	−6.394 (−15.462 to 2.675)	0.162
Age	−0.545 (−1.070—0.019)	0.042	−0.453 (−1.010 to 0.019)	0.032
Parity	2.832(−3.395 to 8.954)	0.359	3.488 (−3.845 to 10.822)	0.341

The results are presented with regression coefficient (B) with 95% confidence interval (CI)

^a^Five of the women were not able to insert the probe because of the pain

^b^Superficial dyspareunia indicates pain in the vaginal opening during sexual intercourse

*NRS* numeric rating scale (0 = no pain and 10 = worst imaginable pain)

**Fig. 2:**
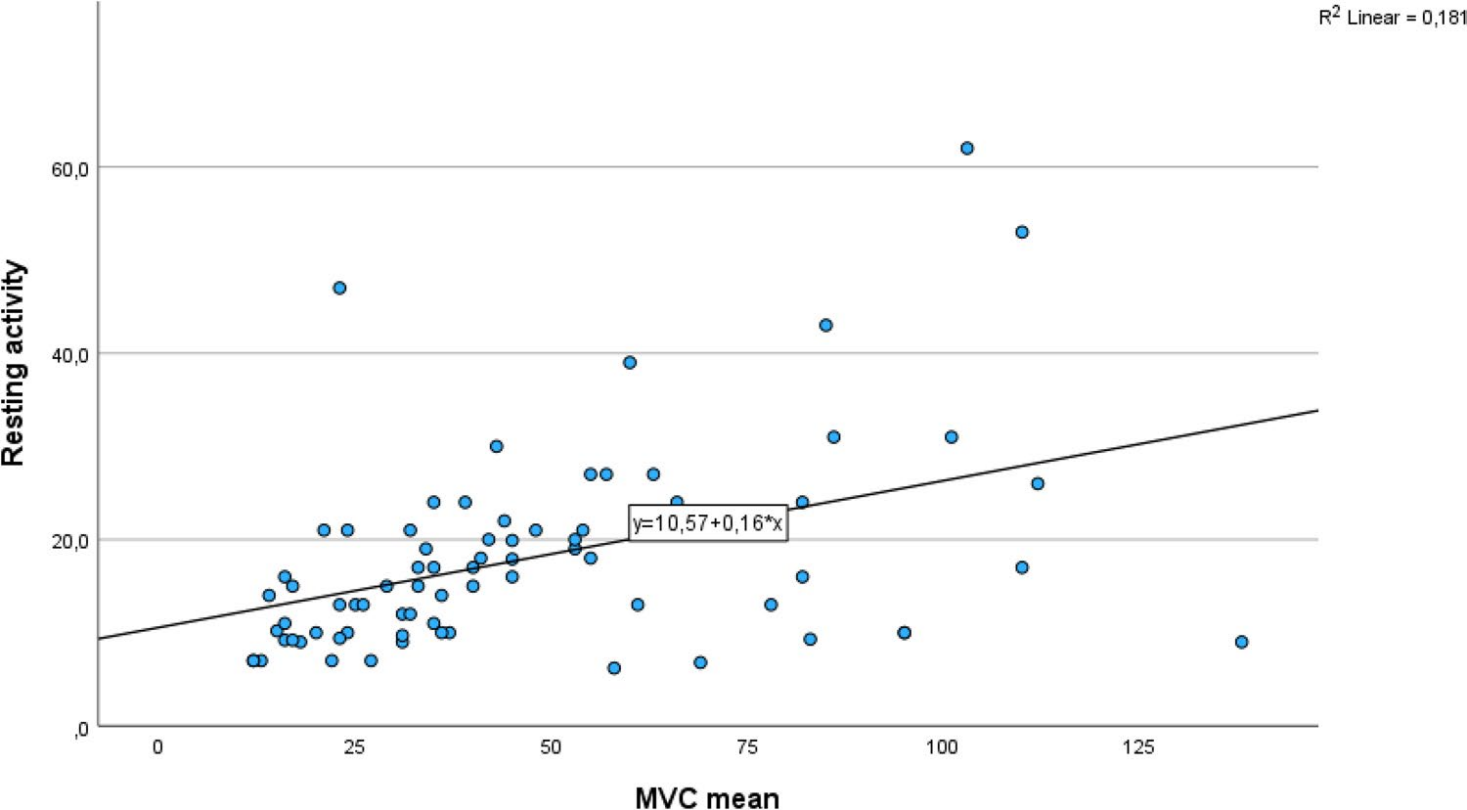
Dot plot illustrating the association between pelvic floor muscle resting activity and maximal voluntary contraction (MVC) measured using surface electromyography (sEMG). The linear regression line (R^2^ = 0.181) follows the equation *Y* = 10.57 + 0.12*X. *n = 75. *Five of the women were not able to insert the probe because of the pain

The unadjusted analysis between PFM resting activity and activation during attempts at PFM MVC was not statistically significant (Table 4). However, controlling for age and parity, a significant relationship was found, indicating a significant positive association between PFM resting activity and activation during attempts at MVCs of the PFM (Table 4). For each unit increase in resting activity, MVC is expected to increase by 0.158 μV (*β* = 158, 95%CI 0.080–0.234, *p* = 0.001; Fig. 2). Further, a negative significant association between PFM resting activity and age (B = −0.425, *p* < 0.05) was found, indicating that resting activity decreases with increasing age, regardless of parity.

**Table 4:** Multiple linear regression analysis showing the association between pelvic floor muscle resting activity and maximal voluntary contraction (MVC) measured with surface electromyography (sEMG). *n* = 75^a^

	Unadjusted estimates		Adjusted estimates	
	B (95% CI)	*p* value	B (95% CI)	*p* value
MVC mean, sEMG µV	0.130 (0.034 to 0.226)	0.009	0.158 (0.080 to 0.234)	0.001
Age	−0.461 (−0.871 to 0.052)	0.028	−0.474 (−3.383 to 6.634)	0.022
Parity	−0.844 (−7.115 to 5.427)	0.789	1.625 (−4.823 to 7.649)	0.654

The results are presented with regression coefficient (B) with 95% confidence interval (CI)

^a^Five of the women were not able to insert the probe because of the pain

## Discussion

This study among women with endometriosis found no significant associations between PFM resting activity, pelvic and genital pain, or location and concerns related to dyspareunia. The results showed, however, an association between resting activity and activation during attempts at MVCs of the PFM measured with sEMG among women with endometriosis. Contrary to the hypothesis, women with increased resting activity could further increase PFM activity during maximal PFM contractions. These results indicate that the ability to contract the PFM is not limited by increased resting activity and could thus be important in future PFM rehabilitation among women with endometriosis.

The study addresses a research area of controversy and brings new insights into the field. The study's strengths include using sEMG, a method that has demonstrated good test–retest and intra- and interrater reliability for assessing resting activity [6]. Further, the PFM measurements were conducted by an experienced physical therapist. However, the use of sEMG has some limitations. The measurements can be influenced by crosstalk from other muscles such as pelvic, hip, and abdominal muscles [21, 22]. In the present study we tried to control for these factors through instruction and feedback by an experienced women’s health and pelvic physical therapist, who observed and supervised the procedure. Further, vaginal lubrication, mucosa, thickness of the vaginal tissue, and movement of the probe can also be a source of error [21, 23]. Other limitations include lack of reference values for increased PFM resting activity, which limits the interpretability of the findings [5]. Another limitation is lack of a control group with no endometriosis, which hampers a direct comparison. Additionally, women with endometriosis often present with multiple comorbidities, further contributing to the complexity of the condition [24]. Establishing clear associations in conditions involving visceral pain and referred pain, such as endometriosis, can be particularly challenging. The phenomenon of referred pain, along with the convergence of somatic and visceral pain pathways, may further obscure direct correlations between PFM activity and perceived pain symptoms [25].

The questionnaire addressing pain location and concerns related to dyspareunia does not encompass all the factors that might impact these conditions. It also lacks psychometric testing, which is a limitation. Thus, our results may be interpreted with caution.

There is limited evidence from studies supporting a clear link between increased resting activity and pelvic or genital pain, and challenges in study design and measurement methods constrain interpretation [5]. Our results correspond with the conclusion of a meta-analysis that found no linear association between pelvic pain and resting activity measured by sEMG [12]. In contrast, a link between increased PFM tone and pain in women with endometriosis has been found in studies utilizing digital palpation [8, 26]. However, palpation does not have psychometric measurement properties as reliable as sEMG and cannot discriminate between active and passive components of PFM tone [3, 9]. Davidson et al. studied 125 physiotherapists testing a novel device that mimics haptic (touch-based) feedback. The device measured three factors: displacement (how much a muscle moves when pressure is applied), force (the amount of pressure applied), and stiffness (the resistance of the muscle to movement). The findings showed that physical therapists could not reliably distinguish between increased and normal PFM tone [9]. This highlights the importance of using measurement methods showing good responsiveness, reliability, and validity in future studies [5].

In the review by Kadah et al., no association between pelvic pain and PFM tone was found. This lack of association may be explained by studies including women with low to moderate pain (> 3 out of 10 on the NRS) [12]. Thus, Kadah et al. recommend that future research should focus on women with moderate to high pain to clarify this relationship [12]. In the present study, where the mean pelvic and genital NRS pain score was 7.5 (indicating severe pain) [27], no association between pain and resting activity was found. However, five women were excluded from the analysis as the sEMG probe could not be inserted because of pain. Although the mean NRS score for these five women was similar to that of the overall sample of 80, it is unclear whether including them would have altered the results.

A negative significant association between PFM resting activity and age indicates that resting activity decreases with increasing age. A decline in muscle activity is expected with age [28]; thus, this finding underscores the importance of controlling for age when examining factors that may affect resting activity. Further, no association with parity was found. However, only nine of the women included were parous, and we may only speculate if the findings would have been different if more parous women had been included [29].

Unfortunately, no reference value for increased or normal PFM tone is currently available. The mean resting activity in this study was 17.5 (SD 16), which clinically is seen as high, but no studies can confirm this assumption [5]. Several studies report increased resting activity in women with provoked vestibulodynia (PVD) compared with a healthy control group, but to our knowledge this relationship has not been investigated among women with endometriosis [21, 30]. However, differences in EMG measurement techniques, such as the use of surface electrodes, various probes, or needle EMG, complicate direct comparisons [31]. It would be important to establish reference values for normal and increased resting sEMG activity in different age groups and populations [32].

The findings from the present study do not support the hypothesis that increased PFM resting activity is associated with reduced activation during MVC. On the contrary, our results indicate a positive correlation between resting activity and activation during attempts at MVCs of the PFM. This result is important for future research on possible treatment approaches among women with endometriosis, where conservative management recommendations are lacking [1, 33]. However, some researchers recommend that women with PVD avoid activities that are assumed to exacerbate their pelvic pain, such as repetitive concentric PFM training, before a “normal” tone is restored [13]. Instead, manual techniques such as connective tissue release in the PFM area, general relaxation, or massage are recommended [34]. In contrast, Naess and Bø found that women with PVD exhibit significantly lower vaginal resting pressure and EMG activity following three maximal PFM contractions [35]. These findings suggest that performing voluntary maximal contractions could be explored as a potential method of reducing increased PFM tone [35]. Thus, this present study emphasizes the necessity for further investigations, as it seems that, despite enduring pain and increased resting activity, women can still to a large degree activate the PFM.

## Conclusion

Contrary to our hypotheses, this study among women with endometriosis, found no association between PFM resting activity and pelvic or genital pain or location, or concerns related to dyspareunia. Further, our results found that higher PFM resting activity resulted in more activation of the PFM during attempts at MVC. Future studies on normative values for PFM resting activity are warranted, as well as whether strength and/or relaxation training of the PFM can influence resting activity and pelvic and genital pain.

## Supplementary Information

Below is the link to the electronic supplementary material.

Supplementary file1 (DOCX 33 KB)

## Data Availability

The data that support the findings of this study are stored in the Tjeneste for Sensitive Data (TSD) at the University of Oslo, Norway. Due to ethical and legal restrictions related to sensitive personal information, the data are not publicly available. Access may be granted upon reasonable request and with necessary approvals from relevant ethical and data protection authorities.
